# Development and validation of a clinical nomogram to predict prostatic inflammation in men with lower urinary tract symptoms

**DOI:** 10.1038/s41391-024-00857-5

**Published:** 2024-07-06

**Authors:** Stavros Gravas, Cosimo De Nunzio, Luís Campos Pinheiro, Javier Ponce de León, Konstantinos Skriapas, Ziad Milad, Riccardo Lombardo, Mariana Medeiros, Pantelis Makrides, Michael Samarinas, Mauro Gacci

**Affiliations:** 1 Medical School, University of Cyprus, Nicosia, Cyprus; 2 Department of Urology, University of Rome, Rome, Italy; 3 Department of Urology, Hospital de São José, Lisbon, Portugal; 4 Department of Urology, de la Fundació Puigvert, Barcelona, Spain; 5 Department of Urology, General Hospital of Larissa, Larissa, Greece; 6 Unit of Urological Robotic Surgery and Renal Transplantation, Careggi Hospital, University of Florence, Florence, Italy; 7 Department of Experimental and Clinical Medicine, University of Florence, Florence, Italy

**Keywords:** Predictive markers, Medical research

## Abstract

**Background:**

Prostatic inflammation is an important etiological component of benign prostatic hyperplasia (BPH) and lower urinary tract symptoms (LUTS). The Prostatic Inflammation Nomogram Study (PINS) aimed to develop and validate a nomogram for predicting the presence of prostatic inflammation in men with LUTS.

**Methods:**

This non-interventional, cross-sectional, prospective study was conducted in six secondary/tertiary centers across Cyprus, Greece, Italy, Portugal, and Spain. Men (≥40 years) with BPH/LUTS scheduled to undergo prostatic surgery or transrectal ultrasound-guided (TRUS) prostate biopsy were included. Fifteen demographic and clinical participant characteristics were selected as possible predictors of prostatic inflammation. The presence of inflammation (according to Irani score) in the prostatic tissue samples obtained from surgery/TRUS biopsy was determined. The effect of each characteristic on the likelihood a prostate specimen demonstrated inflammation (classified by Irani score into two categories, 0–2 [no/minimal inflammation] or 3–6 [moderate/severe inflammation]) was assessed using multiple logistic regression. A nomogram was developed and its discriminatory ability and validity were assessed.

**Results:**

In total, 423 patients (mean age 68.9 years) were recruited. Prostate volume ultrasound (PVUS) > 50 mL, history of urinary tract infection (UTI) treatment, presence of diabetes, and International Prostate Symptom Score (IPPS) Storage score were statistically significant predictors of Irani classification. Logistic regression demonstrated a statistically significant effect for leucocytes detected via urine dipstick, presence of diabetes, PVUS > 50 mL, history of UTIs, and higher IPSS Storage score for the odds of an inflammatory score category of 3–6 versus 0–2. The nomogram had a concordance index of 0.71, and good internal validity.

**Conclusions:**

The nomogram developed from PINS had good predictive ability and identified various characteristics to be predictors of prostatic inflammation. Use of the nomogram may aid in individualizing treatment for LUTS, by identifying individuals who are candidates for therapies targeting prostatic inflammation.

## Introduction

The role of prostatic inflammation in the development and progression of benign prostatic hyperplasia (BPH) and the severity of associated lower urinary tract symptoms (LUTS) is increasingly being recognized [1, 2]. As a result, prostatic inflammation has become a target for the treatment of LUTS [3, 4].

To optimize medical strategies for the management of LUTS, it is important to identify patients who may have prostatic inflammation. One possible approach is the use of biomarkers (clinical and/or laboratory parameters) to identify such inflammation. Given the influence of inflammation on prostate-related conditions, there is a pressing need to explore and devise new biomarkers or imaging techniques for detecting prostate inflammation and monitoring its progress post-treatment. Up to this point, the definitive method for diagnosing prostate inflammation and determining its severity and spread has relied on examining tissue samples obtained through prostate biopsies, radical or simple prostatectomies, or transurethral resection of the prostate (TURP); however, a less invasive tool would be clinically beneficial. To address these challenges, various studies have suggested new biomarkers found in serum, urine and seminal plasma (such as C-reactive protein, MPC-1, inducible costimulator, interleukin [IL]-6, IL-8, IL-10, tumor necrosis factor-alpha, zinc levels and presepsin) to estimate the presence and intensity of chronic inflammation [2–5]. However, none of these markers have been confirmed as definitive indicators of prostate inflammation or have been adopted in clinical settings. Therefore, it remains crucial to develop a reliable method for identifying individuals at risk for prostatic inflammatory infiltrates [2].

Chronic inflammation, caused by infections, exposure to environmental factors, or a combination of both, plays a role in the development of about 20% of human cancers, including those of the stomach, liver and large intestine. Studies of epidemiology, tissue pathology and molecular biology are increasingly suggesting that inflammation of the prostate may play a key role in the development and advancement of prostate cancer. Genes linked to prostate cancer susceptibility, including *RNASEL*, *MSR1* and *MIC1*, found in areas associated with familial prostate cancer, as well as *TLR4*, *MIC1*, *PON1*, *BRCA2*, *CHEK2* and *OGG*, have been identified as contributors to prostate cancer development [2–5]. Many of these genes are responsible for encoding proteins essential in the body’s defense against infection, inflammation, and oxidative stress. Mutations in these genes may impair the body’s ability to prevent cancer through this route. Prostatic intraepithelial atrophy, which is often linked to inflammation in the prostate, is seen as a potential early stage of high-grade prostatic intraepithelial neoplasia and prostate cancer. These atrophy lesions are commonly found on the prostate’s periphery and are thought to result from the regenerative proliferation of prostate epithelial cells in response to injuries caused by infection or oxidative damage to cells [2–5].

The aim of the Prostatic Inflammation Nomogram Study (PINS) was to develop and validate a nomogram that could be used to predict the presence of prostatic inflammation in men with LUTS.

## Materials and methods

### Study design and objectives

PINS (ClinicalTrials.gov, NCT04856748) was a non-interventional, multicenter, cross-sectional, observational, prospective study. It was conducted in six secondary and tertiary centers in five southern European countries (Cyprus, Greece, Italy, Portugal, and Spain).

The study was conducted according to the principles of the Declaration of Helsinki (2013 version) and in accordance with the International Conference on Harmonization’s standards for Good Clinical Practice. The protocol of the study and all necessary documentation was approved by the institutional review boards and ethics committees of the participating hospitals. All patients provided written informed consent before study participation.

### Participants

Eligible patients were men, aged ≥40 years, with BPH and LUTS who were scheduled to undergo any prostatic surgery for benign prostatic obstruction (BPO; including open, laparoscopic, robotic transurethral resection/enucleation, or laser prostatectomy) or transrectal ultrasound-guided prostate biopsy (TRUS-biopsy), according to the standard clinical practice of their treating physician.

Exclusion criteria were treatment with any plant extract or 5α-reductase inhibitors during the previous 3 months; a history of pelvic radiotherapy; a history of prostatectomy or transurethral resection of a bladder tumor or previous TRUS-biopsy; presence of an indwelling catheter; prostate cancer found at the biopsy; no LUTS (International Prostate Symptom Score [IPSS] [6] of 0); or the lack of a prostate specimen (vaporization of the prostate).

### Study procedures

Baseline demographic and clinical characteristics of the participants considered to be possible predictors of prostatic inflammation were recorded using a case report form prior to prostatic surgery or TRUS-biopsy. Fifteen characteristics were considered.

Prostatic tissue samples obtained from the prostatic surgery or TRUS-biopsy underwent a standard pathological examination and inflammation was determined according to the Irani score (total score, and histologic inflammation grading and aggressiveness grading sub-scores). The Irani scoring system uses a 4-point scale for inflammation (0 = no inflammatory cells, 1 = scattered inflammatory cell infiltrate, 2 = nonconfluent lymphoid nodules, 3 = large inflammatory areas with confluence of infiltrate) and aggressiveness (0 = no contact between inflammatory cells and glandular epithelium, 1 = contact between inflammatory cell infiltrate and glandular epithelium, 2 = clear but limited, i.e. <25% of examined material, glandular epithelium disruption, 3 = glandular epithelium disruption on ≥25% of examined material) [7].

### Statistical analysis

The planned sample size was 375 patients based on the recommendation for nomogram development that the minimum value of the frequencies of two response levels should be greater than 10 times the number of predictors when the outcome is binary (i.e., the presence of prostatic inflammation being “yes” or “no”) [8]. The aim was to include the 15 characteristics (candidate predictors) in the nomogram. Assuming that the incidence of prostatic inflammation in the biopsies would be around 60% [9, 10], it was calculated that 150 patients would be needed in the non-inflammation group and 225 patients in the inflammation group.

The demographic and clinical candidate predictors that were categorical variables were expressed as frequencies and percentages, while those that were continuous variables (such as age, maximum urinary flow [Qmax] and IPSS total score) were described as mean ± standard deviation (SD), with the respective number of observations in each case.

The baseline demographic and clinical candidate predictors and the histological outcome (i.e., presence of inflammation measured by the Irani score) of the prostate specimens were used to develop the nomogram. Total Irani score was classified into two categories, scores of 0–2, representing no/minimal inflammation, and scores of 3–6, representing moderate/severe inflammation. The effect of each predictor on the binary score classification was examined through univariate analysis and all predictors with a level of statistical significance of 0.2 were included in a multiple logistic regression model. The multiple logistic regression model was applied to assess the statistical significance and independence of the prognostic predictors; patient age was also included to enhance the generalizability of the nomogram. A backward method was applied to check for differences in the models. Interactions between statistically significant predictors were examined, with no statistical significance identified. Final pruning included examining the effect of all formerly non-significant predictors.

The discriminatory ability of the nomogram was assessed using a receiver operating characteristic (ROC) analysis to determine the concordance index (C-index). Internal validation was conducted using split-sample validation [11, 12]. In this method, cross validation on 10 “folds,” or groups of approximately equal size, was performed to assess the validity of the nomogram. The first fold was treated as a validation set and the nomogram was fitted on the remaining 9 folds. Calibration plots were produced to determine the internal validity of the nomogram.

Statistical analysis was performed using Orange software (version 3.33.0) [13], and significance was set at 0.05 in all cases.

## Results

### Study population

The study began in September 2020; the primary completion date was September 2022. In total, 423 patients were recruited, with a mean age (SD) of 68.9 ± 8.1 years. Of these patients, 293 (69.3%) had undergone prostatectomy and 130 (30.7%) had undergone TRUS-biopsy.

Further baseline characteristics are provided in Table 1.

**Table 1:** Baseline demographic and clinical characteristics of the study participants.

Characteristic	Results^a^	Number of patients
Age, years	68.9 ± 8.1	423
IPSS Total score	14.6 ± 7.1	423
IPSS Voiding score	7.3 ± 4.6	423
IPSS Storage score	6.9 ± 3.7	423
Body mass index, kg/m^2^	26.4 ± 3.7	423
PVUS, mL^b^	70.9 ± 32.6	423
Qmax, mL/sec	10.9 ± 4.5	420
PSA, ng/mL	6.1 ± 6.5	421
Post-void residual volume, mL	69.7 ± 56.7	315
Previous or current medication for LUTS/BPH (yes)	301 (71.3%)	422
Metabolic syndrome (yes)^c^	91 (21.7%)	419
Presence of calcifications (yes)^b^	165 (39.1%)	422
Diabetes (yes)^d^	60 (14.2%)	423
Urine dipstick positive for leucocytes (yes)	76 (18.2%)	417
History of confirmed UTIs (yes)	64 (15.2%)	421

*BPH* benign prostatic hyperplasia, *IPSS* International Prostate Symptom Score, *LUTS* lower urinary tract symptoms, *PSA* prostate-specific antigen level, *PVUS* prostate volume ultrasound, *Qmax* maximum urinary flow, *UTIs* urinary tract infections.

^a^Categorical and continuous variables are expressed as percentages and mean ± standard deviation, respectively.

^b^Assessed using transrectal or abdominal ultrasound.

^c^Metabolic syndrome was defined using the USA National Cholesterol Education Program – Adult Treatment Panel III (NCEP-ATPIII) [44], with the following components considered – waist circumference, triglyceride level, blood pressure, fasting glucose level, high-density lipoprotein cholesterol level, or any treatment for these components. Patients with known diabetes and/or hypercholesteremia and/or arterial hypertension under treatment were considered to be positive for the specific component.

^d^Determined from patient and drug prescription history, and serum glucose levels in patients without known diabetes.

### Nomogram

Table 2 presents the univariate and multivariate analysis of all the tested parameters.

**Table 2:** Univariate and multivariate analysis of all the tested parameters.

Parameter	Univariate analysis		Multivariate analysis	
	OR (95% CI)	*P* value	OR (95% CI)	*P* value
Presence of calcifications	0.771 (0.481–1.234)	0.277	0.767 (0.451–1.301)	0.325
Urine dipstick positive for leucocytes	6.665 (2.369– 8.752)	<0.001	**6.018 (2.101–17.239)**	**0.001**
PSA, ng/mL	0.973 (0.943–1.005)	0.094	0.968 (0.931–1.006)	0.100
Post-void residual volume, mL^a^	1.003 (0.998–1.007)	0.222	–	–
Metabolic syndrome	0.752 (0.424–1.334)	0.329	0.702 (0.302–1.635)	0.412
Presence of diabetes mellitus	2.175 (0.996–4.750)	0.049	**2.066 (0.912–4.682)**	**0.028**
Previous or current medication for LUTS/BPH	0.577 (0.358–0.932)	0.023	0.882 (0.497–1.565)	0.668
History of confirmed UTIs	4.029 (1.568–10.354)	0.002	**3.113 (1.172–8.270)**	**0.023**
IPSS Total score	1.049 (1.014–1.086)	0.006	1.012 (0.951–1.077)	0.708
IPSS Storage score	1.092 (1.024–1.163)	0.007	**1.077 (1.008–1.152)**	**0.029**
PVUS, mL	2.017 (1.268–3.209)	0.003	**1.922 (1.174–3.146)**	**0.009**
Qmax, mL/sec	0.968 (0.921–1.017)	0.192	0.986 (0.931–1.044)	0.622
Age, years	1.014 (0.986–1.042)	0.329	1.001 (0.972–1.031)	0.943
Body mass index	1.024 (0.962–1.091)	0.453	0.983 (0.905–1.066)	0.674

Parameters listed in Table 2 are candidates predictors of the prostatic inflammation. Values in bold highlight the parameters where a statistically significant effect was observed after applying the statistical model described in the Methods section.

*BPH* benign prostatic hyperplasia, *CI* confidence interval, *IPSS* International Prostate Symptom Score, *LUTS* lower urinary tract symptoms, *OR* odds ratio, *PSA* prostate-specific antigen level, *PVUS* prostate volume ultrasound, *Qmax* maximum urinary flow, *UTIs* urinary tract infections.

^a^Not included in the multivariate analysis due to the smaller number of patients (*n* = 315).

The logistic regression model of the odds of an inflammation score category of 3–6 versus 0–2 identified a statistically significant effect regarding leucocytes detected via urine dipstick (odds ratio [OR] 6.02, 95% confidence interval [CI] 2.10–17.24; *p* = 0.001), prostate volume >50 mL (OR 1.92, 95% CI 1.17–3.15; *p* = 0.009), history of urinary tract infections (UTIs; OR 3.11, 95% CI 1.17–8.27; *p* = 0.023), presence of diabetes mellitus (DM; OR 2.07 95% CI 0.92–4.68; *p* = 0.028), and higher IPSS Storage score (OR 1.08, 95% CI 1.01–1.15; *p* = 0.029). A 2D projection approach was adopted for the visualization of IPSS Storage and age.

**Fig. 1:**
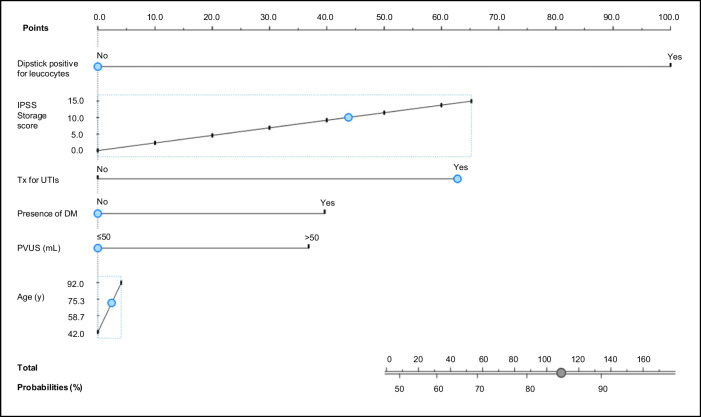
Nomogram for the prediction of an inflammation score category of 3–6 versus 0–2. IPSS International Prostate Symptom Score, PVUS prostate volume ultrasound, Tx treatment, UTIs urinary tract infections.

Based on the results of the logistic regression model, a nomogram was generated (Fig. 1). For each variable in the nomogram, a number of points was assigned to a given magnitude of the variable according to a points scale; the cumulative points score was then summed for all variables to give the probability of prostatic inflammation. The nomogram estimated the probability of a classification into an inflammation score category of 3–6 versus 0–2 for each patient on the basis of their results on each candidate predictor.

#### Discriminatory ability and validation

**Fig. 2:**
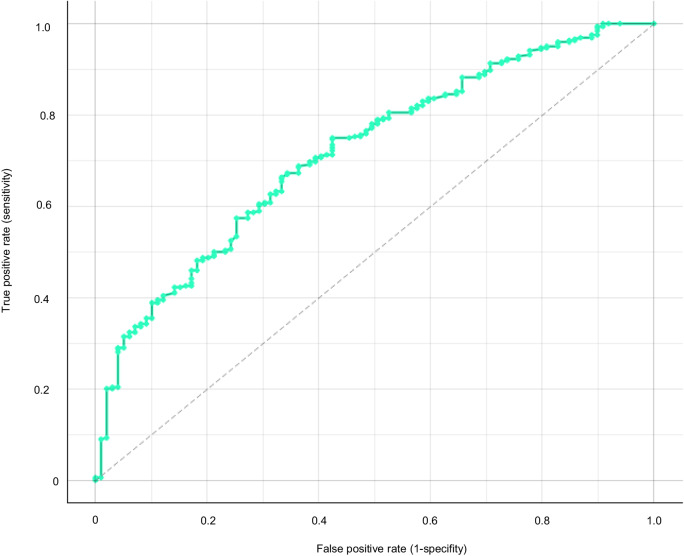
Receiver operating characteristic curve for the nomogram.

The area under the curve of the nomogram (the C-index) was 0.71, and calibration plots showed slight deviation from the main diagonal (Fig. 2). The split-sample validation showed that the nomogram had acceptable internal validity.

## Discussion

The nomogram developed from PINS incorporates certain clinical characteristics as predictors of prostatic inflammation, namely leucocytes identified on the urine dipstick test, prostate volume >50 mL, history of UTIs, presence of DM, and higher IPSS Storage score. The C-index (0.71) indicates that the nomogram had good predictive/diagnostic accuracy.

The findings from PINS and the nomogram developed from its data are in line with the available literature. In our nomogram, metabolic syndrome was not a predictor of prostatic inflammation, probably because of the low prevalence of metabolic syndrome in the individuals included this study (21.7%) compared with other studies [9]. However, DM was predictive of prostatic inflammation, as observed in other trials [14]. The nomogram also indicated that previous treatment of UTIs was predictive of prostatic inflammation, suggesting that a previous infection may drive this inflammation. Further, the fact that a positive urine dipstick test for leucocytes was also a predictor of prostatic inflammation suggests the involvement of current infection in the etiology of the inflammation. Among the other predictors identified by the nomogram, prostate volume has been previously reported to be positively associated with prostatic inflammation [15–19], although the association is sometimes reported to be weak [15, 17]. Further, the nomogram indicated that higher IPSS Storage score was a predictor of prostatic inflammation, which is consistent with findings from other studies that have noted a positive association between markers of such inflammation, particularly chronic inflammation [17], and IPSS storage symptoms [20–23].

The presence of prostatic inflammation is clinically important, as it has been associated with more severe disease [18, 24, 25] and worse treatment outcomes [26, 27]. Recently, Cash et al. have proposed an interesting physio-pathological mechanism behind Marion disease (contracture of the bladder neck). Overall, chronic prostatic inflammation could lead to the deposition of collagen fibers causing dynamic changes and resulting in bladder outlet obstruction. Such data and theory clearly suggest that patients with prostatic inflammation should be identified promptly and treated accordingly to avoid histological changes [28]. While the impact of drug treatments for BPH/LUTS on prostatic inflammation has not been fully elucidated, there are clinical data to indicate that some treatments that are effective in managing LUTS have anti-inflammatory effects (including an extract of *Serenoa repens* [29–31], tamsulosin [29], and tadalafil and vardenafil [32]) and that established anti-inflammatory agents, such as cyclooxygenase inhibitors [33] and non-steroidal anti-inflammatory drugs (NSAIDs) [34], may improve LUTS, and possibly prevent or delay the development of BPH [35].

Given the availability of agents with anti-inflammatory effects that are effective for LUTS, more accurate stratification of patients for whom such treatment would be beneficial is important. Currently, prostatic inflammation is identified only by prostate biopsy and reported as a secondary finding; while providing definitive results, such biopsy is invasive, costly, and only indicated when prostate cancer is suspected. Further, the use of serum, urine and seminal biomarkers to identify prostatic inflammation are still under investigation. Therefore, our nomogram can overcome the actual unmet needs in prostatic inflammation identification.

In particular, the implementation of the nomogram in clinical practice may improve the management of patients with prostatic diseases. Overall, LUTS/BPH medical treatment has several different targets, including α-adrenergic receptors, 5α-reductase, phosphodiesterase type 5 and inflammation [36]. Hypothetically, patients with a high probability of inflammation may be treated with drugs with anti-inflammatory effects, such as the hexanic extract of *Serenoa repens* [37]. In the past few years, several authors have suggested tailoring medical treatment to patients based on the physiopathology of their disease [38]. The predictive nomogram developed from PINS has the potential to form part of such an individualized approach. Several studies have recently considered prostatic inflammation as a new target for LUTS/BPH prevention and treating strategies [39]. Patients with prostatic inflammation also experience different outcomes after medical and surgical treatment [39]. Identifying patients at high risk of prostatic inflammation may improve patient counseling before medical or surgical treatment. Although our nomogram should be validated in other studies before its implementation in clinical practice, it represents an easy-to-use tool to identify patients at risk of prostatic inflammatory infiltrates defined according to the Irani score.

There are, however, a number of limitations that need to be considered. Firstly, this nomogram aims to identify individuals with moderate/severe prostatic inflammation, identified using the Irani score and the results cannot be generalized to other classifications such as the inflammatory score. Moreover, prostate specimen examination was not centralized and, thus, there may have been inconsistency in the grading of inflammation across laboratories. Another possible limitation is that patients did not perform the Meares Stamey test. However, this test is indicated for the evaluation of acute prostatitis and is not the standard for the evaluation of grade and aggressiveness of prostatic inflammatory infiltrates. A further limitation is the lack of questionnaires, such as the National Institutes of Health-Chronic Prostatitis Symptom Index (NIH-CPSI) questionnaire; however, in the initial protocol of our study this was not considered. We acknowledge that the NIH-CPSI is the standard questionnaire to evaluate patients with prostatitis, but it has not been used in studies evaluating inflammatory infiltrates in patients with LUTS/BPH or in patients with metabolic syndrome [9, 40–43].

In conclusion, this paper describes for the first time a nomogram that is an easy-to-use, noninvasive method to predict prostatic inflammation in men with LUTS. Indeed, the PINS nomogram is the first and only nomogram available for this purpose. It incorporates clinical biomarkers that are quick and inexpensive to obtain in daily clinical practice, and familiar to urologists. If externally validated, our nomogram may aid in identifying patients with moderate/severe prostatic inflammation and who are suitable candidates for therapies targeting prostatic inflammation.

## Data Availability

The datasets used and analyzed during the current study are available from the corresponding author on reasonable request.
